# Supplementary material for: The maize gene maternal derepression of r1 encodes a DNA glycosylase that demethylates DNA and reduces siRNA expression in the endosperm
Source: Plant Cell. 2022 Jul 1;34(10):3685–701. doi: 10.1093/plcell/koac199 (PMC9516051; doi:10.1093/plcell/koac199)
Supplement: koac199_Supplementary_Data [file koac199_supplementary_data.zip › koac199_Supplementary_Data/tpc.21.00848_Supplemental_Figures and Tables.pdf]

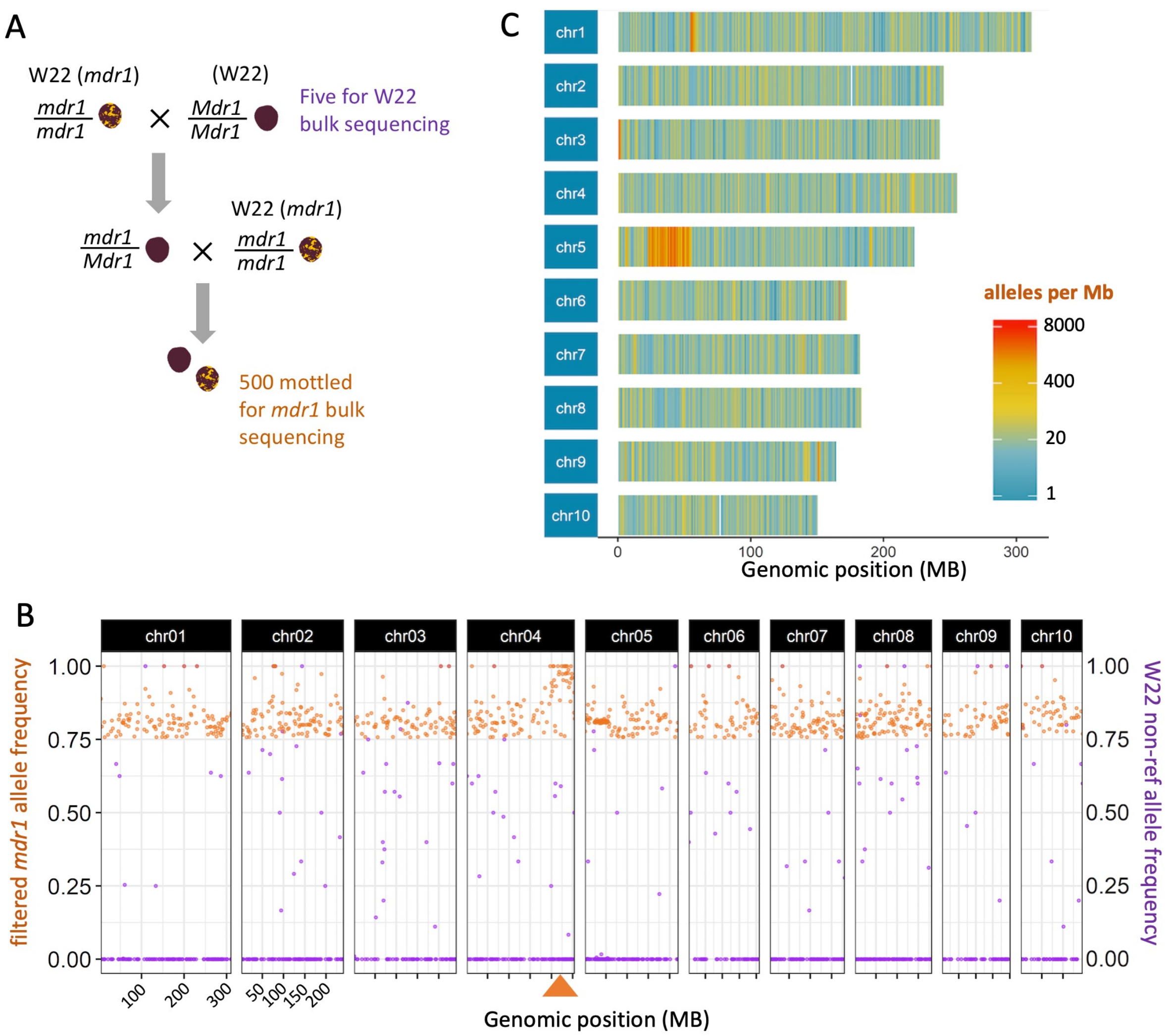

Supplemental Figure S1 (supports Figure 1)
A, Schematic depiction of *mdr1* mapping method based on kernel mottling phenotype.
B, Allele frequency is the number of reads supporting the non-W22-reference allele divided by the total number of reads at that site, averaged over 1-Mb intervals. The left Y-axis (orange) indicates variant allele frequencies for the *mdr1* bulk, filtered to only include ones with a frequency greater than 0.75. Values are averaged over non-overlapping 1 Mb intervals. According to the crossing scheme (see panel A), *mdr1*-linked alleles should approach frequencies of 1, while unlinked ones should average 0.75. The right Y-axis (purple) indicates allele frequency for the W22 bulk for all variant alelles, not just ones with a frequency greater than 0.75. The X-axis indicates genomic position, with major tick marks on 50-Mb intervals, except for chromosome 1 on 100-Mb intervals. The arrowhead on the distal tip of chromosome 4 indicates position of *dng101*/*mdr1*.
C, Chromosome heatmaps indicate the number of non-W22-reference alleles per Mb in the *mdr1* bulk. High frequency alleles reveal regions that are not shared between the sequenced W22 stock and Kermicles's *mdr1* W22 stock. These regions do not include the *mdr1* region on the tip of 4L.

maternal genotype		paternal genotype	viable progeny that inherited both mutations	
Mdr1/*Mdr1* *Dng102*/*Dng102*	X	*Mdr1*/*mdr1* *Dng102*/*mu1083641*	0 of 35	(P-value = 0.0003)
Mdr1/*mdr1* *Dng102*/*mu1083641*	X	*Mdr1*/*Mdr1* *Dng102*/*Dng102*	0 of 72	(P-value < 0.0001)
Mdr1/*Mdr1* *Dng102*/*Dng102*	X	*Mdr1*/*mdr1* *Dng102*/*dng102-Q235*	0 of 23	(P-value = 0.0028)
Mdr1/*mdr1* *Dng102*/*dng102-Q235*	X	*Mdr1*/*Mdr1* *Dng102*/*Dng102*	0 of 46	(P-value < 0.0001)

Supplemental Figure S2 (supports Figure 2)
Crosses between heterozygous double mutant *mdr1 dng102* and wild-type fail to transmit both mutations simultaneously. Kernels from each cross were planted and seedling leaves genotyped by PCR. P-values are based on a Fisher's exact test compared to the expected frequency of 25% double mutant transmission.

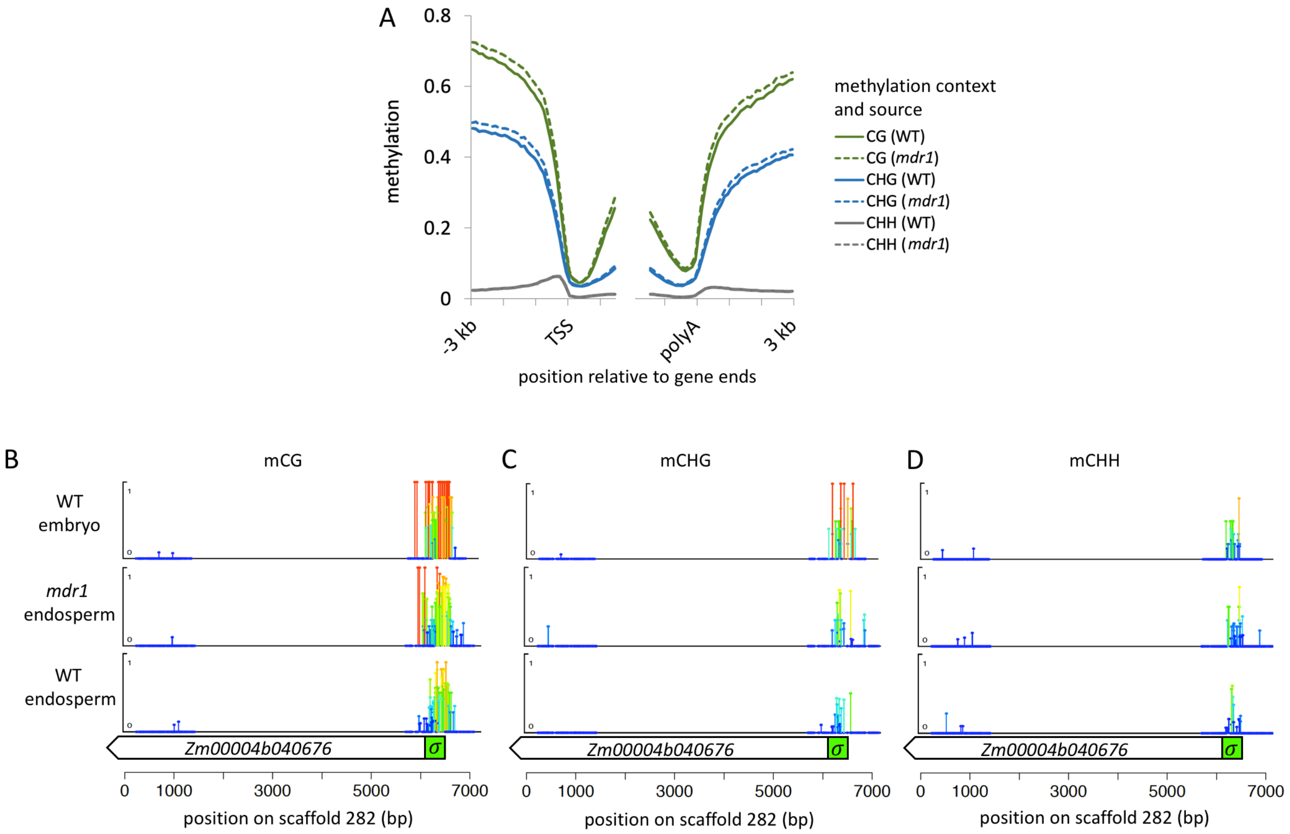

Supplemental Figure S3 (supports Figure 3)
A, Endosperm wild-type and *mdr1* mutant mCG, mCHG, and mCHH profiles relative to gene ends. Methylation values are averages of 100-bp intervals. TSS = Transcription start site, polyA = polyadenylation site.
B, Differential methylation of an *r1* gene in embryo, endosperm, and *mdr1* mutant endosperm, same as Figure 3A. mCG values are shown at single-base level resolution over an 8-kb region including the majority of an *r1* homolog in W22 (*Zm00004b040676*). Both height and color of lollipops indicate mCG values (darkest blue = zero mCG). Regions without lollipops indicate lack of read coverage, either because of multi-copy sequence or large N gaps.
C, Same as B, but mCHG.
D, Same as A, but mCHH.

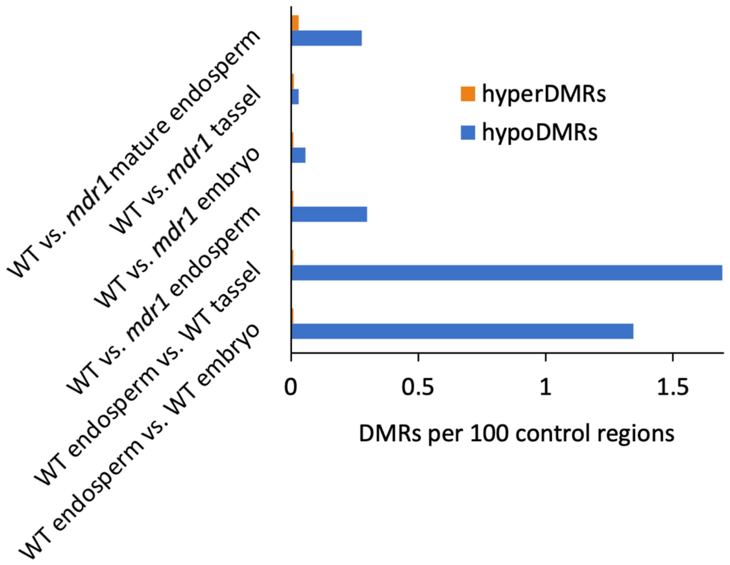

Supplemental Figure S4 (supports Figure 3)
HypoDMR and hyperDMR frequencies for pairwise comparisons of multiple tissues and genotypes. Control regions are the 200-bp regions that were eligible for identifying DMRs based on read coverage and number of informative cytosines. Mature endosperm is from dry seeds. Other endosperm samples are 15-DAP. Embryos are also 15-DAP. Tassel is premeiotic stage.

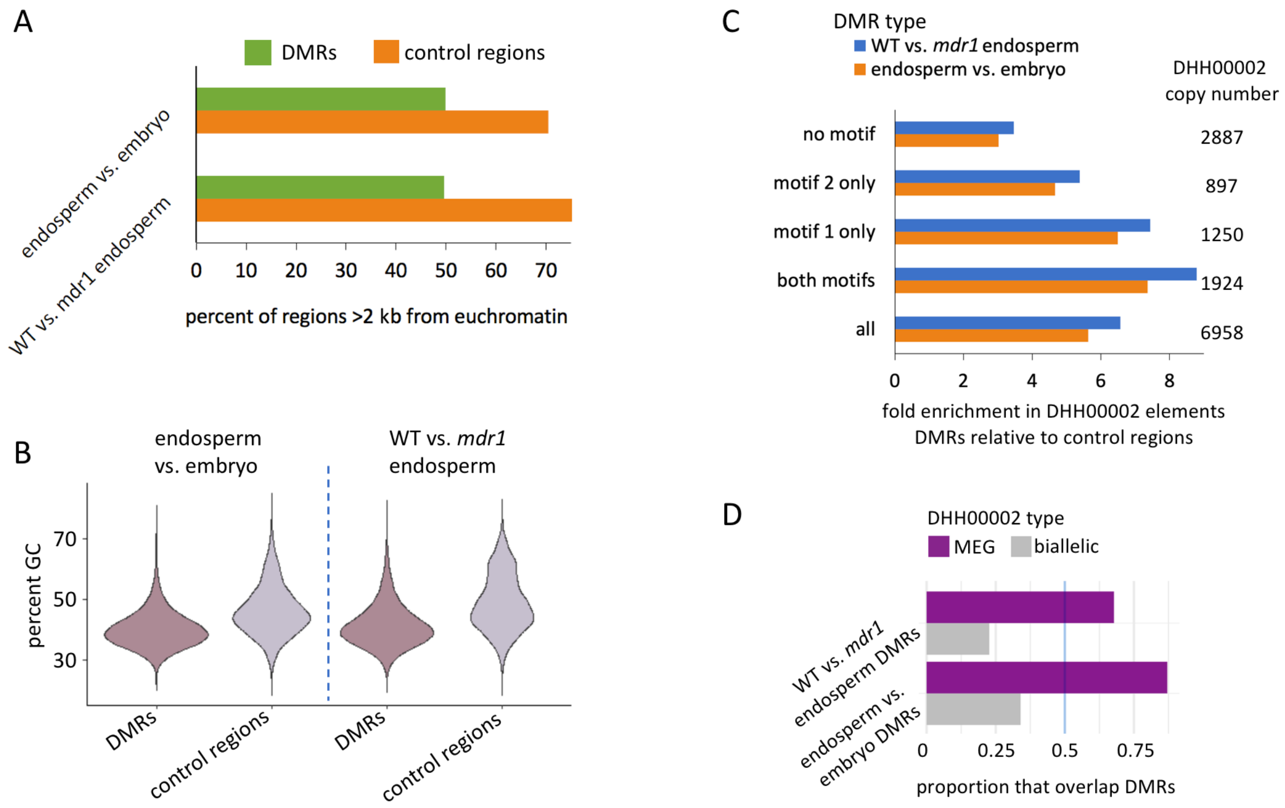

Supplemental Figure S5 (supports Figure 3)
A, Percent of regions that are greater than 2 kb from nearest euchromatin. Euchromatin is defined by 200 bp regions with mCHG < 0.2 in 15-DAP embryo.
B, GC content of DMRs and control regions.
C, DMR enrichment in Helitron family DHH00002. Enrichment is the proportion of DMRs that overlap at least 50% of their length (100 bp) with DHH00002 elements divided by the proportion of control regions that do. Control regions are the 200-bp regions that were eligible for identifying DMRs based on read coverage and number of informative cytosines. Motif 1 is TTWCCATATW and motif 2 is CCAWAAATGG.
D, Maternal imprinted DHH00002 Helitrons have a high frequency of overlap with endosperm DMRs. MEG-type DHH00002 elements (31 elements) have a maternal ratio value greater than 0.9 and non-imprinted (150 elements) have a maternal ratio between 0.1 and 0.9. The Y-axis indicates the proportion of elements that overlap endosperm DMRs from each of the two comparisons. In both, MEG-type DHH00002 elements are significantly enriched for DMRs over non-imprinted ones (p-value < 0.01, binomial test).

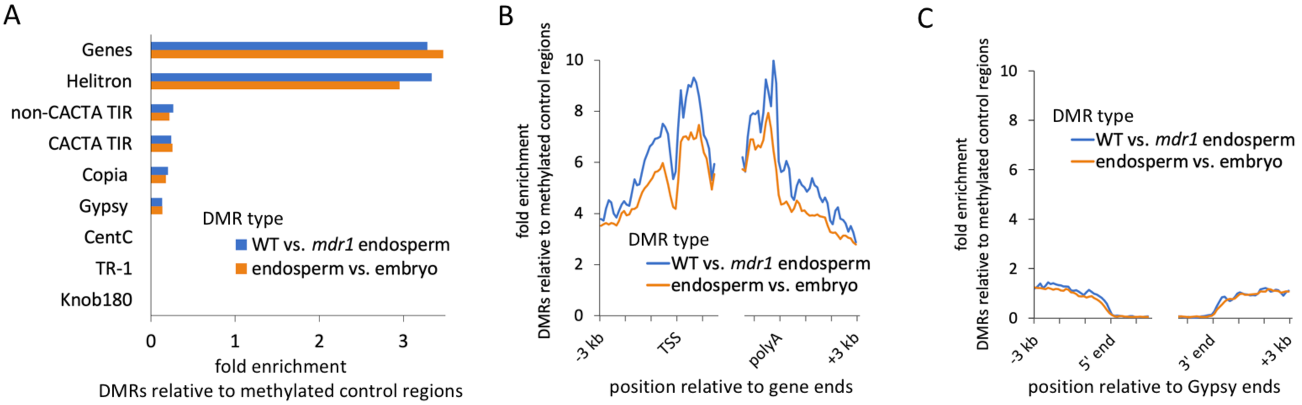

Supplmental Figure S6 (supports Figure 3)
A, DMR enrichment relative to methylated control regions in different genetic elements. Enrichment is the proportion of DMRs that overlap at least 50% of their length (100 bp) divided by the proportion of methylated control regions that do. Methylated control regions are the 200-bp eligible regions used to define DMRs, but only including the subset where both mCG and mCHG exceeded values of 0.2 in at least one of the two methylomes.
B,C, Enrichment for DMRs relative to methylated control regions on 100-bp intervals relative to gene ends (C) or Gypsy ends (D). Gypsies with solo LTRs were excluded from this analysis. For genes, TSS = transcription start site, polyA = polyadenylation site.

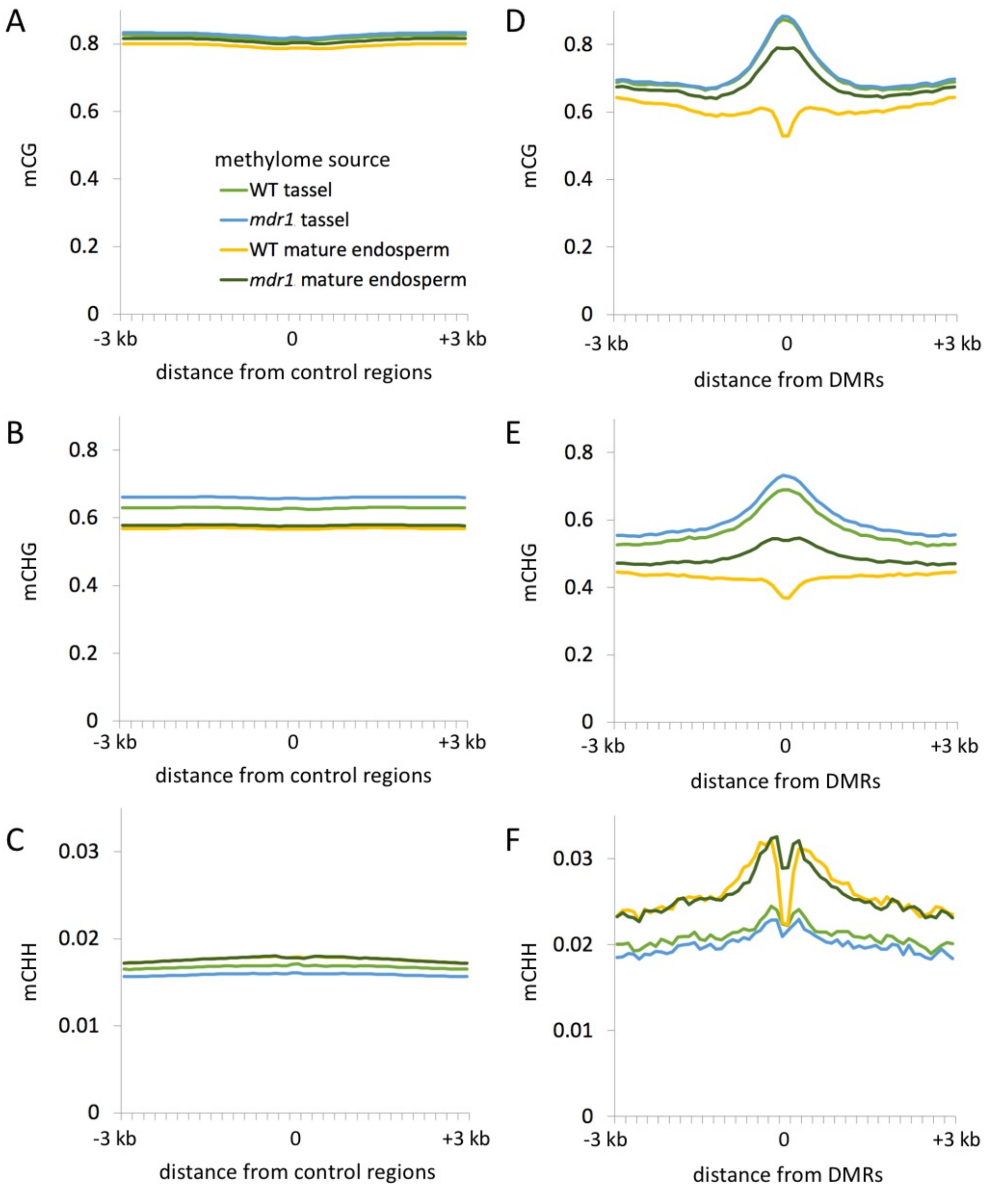

Supplemental Figure S7 (supports Figure 4)
A-C, mCG, mCHG, and mCHH profiles centered on control regions associated with wild-type vs. *mdr1* endosperm DMRs. Methylation values are averages of 100-bp intervals within and up to 3 kb on either side of each 200-bp region. Mature endosperm is from dry seeds. Tassel is premeiotic stage.
D-F, Same as A-C, except methylation centered on wild-type vs. *mdr1* DMRs instead of control regions.

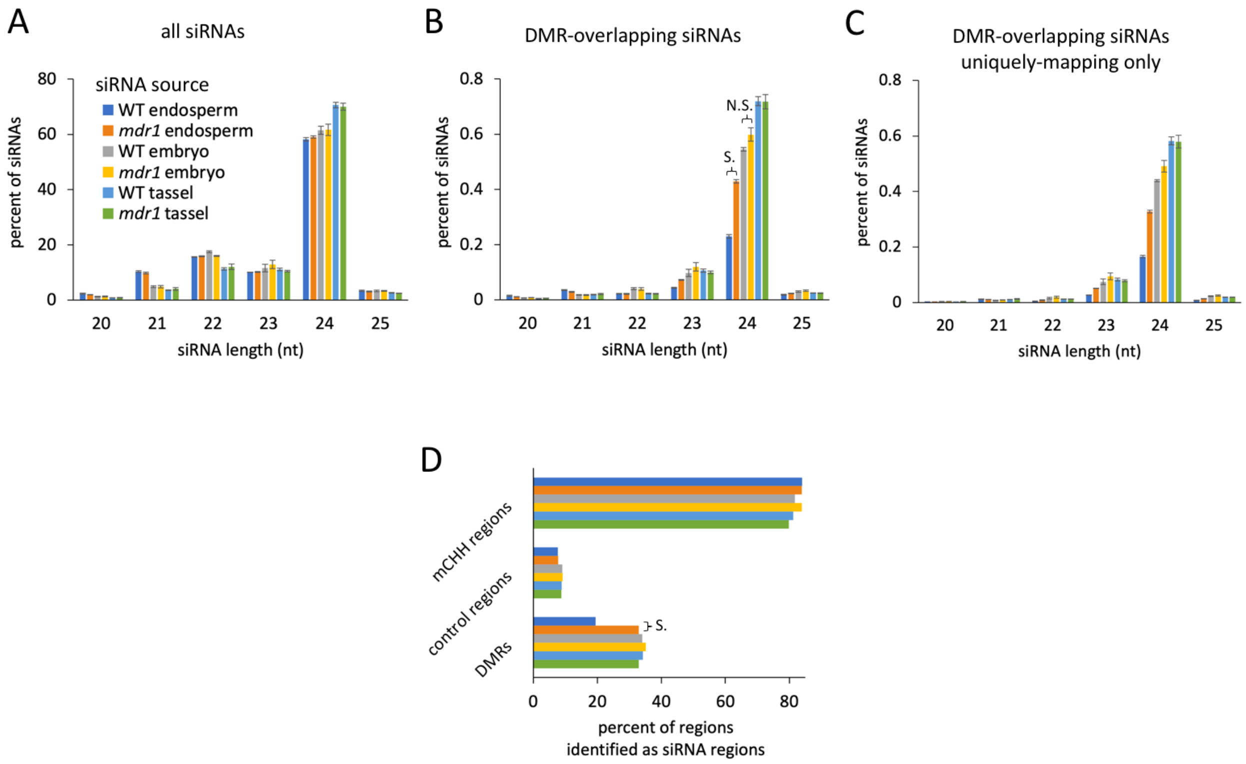

Supplemental Figure S8 (supports Figure 4)
A, Length distributions of all siRNAs. Error bars are standard errors of the means for biological replicates. Same as Figure 4G, except including premeiotic stage tassel siRNAs.
B, As in A, except only siRNAs that overlapped at least 90% of their length with wild-type vs. mdr1 endosperm DMRs. “S.” indicates significant difference: P-value < .00001, two-tailed Student t-test. “N.S.” indicates not significant: P-value = .06. Same as Figure 4H, except including premeiotic stage tassel siRNAs.
C, As in B, but only including uniquely-mapping siRNAs.
D, Regions categorized by whether they produce siRNAs. An “siRNA region” is any that had at least 50 bp spanned by siRNAs of the indicated source tissues. All libraries were subsampled to 20 million siRNA reads. DMRs and control regions are from the wild-type vs. mdr1 endosperm comparison. mCHH regions are 200-bp regions with an average mCHH value of at least 0.2 in wild-type endosperm. Same as Figure 4I, except including premeiotic stage tassel siRNAs. “S.” indicates significant difference (P-value < .0001 two-tailed chi-square with Yates correction).

Table S1: Number of regions overlapping different genetic elements

	control regions		DMRs	
	WT vs. *mdr1* endosperm	endosperm vs. embryo	WT vs. *mdr1* endosperm	endosperm vs. embryo
total region count	6169832	3938072	18464	52919
CACTA (DTC)	19899	13154	35	115
CentC	221	164	0	0
Copia (RLC)	1442009	833117	974	2307
Gypsy (RLG)	2771287	1524039	1243	3217
Harbinger (DTH)	51557	31466	98	270
hAT (DTA)	13214	9024	74	166
Helitron (DHH)	305191	211835	2803	7768
Knob180	4839	4370	0	0
Mariner	43675	31326	78	249
MEG	466	382	22	73
Mutator (DTM)	1472	1013	14	25
PEG	1275	1098	2	15
LINE (RIL)	6635	4837	22	65
LINE (RIT)	1413	1019	5	13
SINE (RST)	947	659	6	19
Gene	390963	310434	1016	3346
TR-1	352	317	0	0
unclassified LTR retrotransposon (RLX)	702233	441015	1715	4575

Other than total region counts, the numbers in the table indicate the number of differentially methylated regions (DMRs) or control regions that overlap at least 50% of their length (100 bp) with each of the indicated genetic elements. Control regions are the 200-bp regions that were eligible to be DMRs based on read coverage and number of informative cytosines. DMRs had reduced methylation in wild-type endosperm relative to either mdr1 mutant endosperm (WT vs. *mdr1* endosperm) or to wild-type embryo (endosperm vs. embryo). Only genes that are annotated in both the B73 v4 genome and W22 v2 genome assemblies were included in the analysis. MEGs and PEGs are the subset of genes that are imprinted, as defined in Anderson et al. (2021) Widespread imprinting of transposable elements and variable genes in the maize endosperm. PLoS Genetics.

Table S2: Primer sequences

name	sequence
W22-Ros1-F1	TTAGAACAAGGCGACTTTTCACC
W22-Ros1-R1	AGGAACTCGGTAGCTCTTCT
JIG-339	TCCAAAGCCTCCCAAAGAAA
JIG-340	GCATATGATCGACCGAGCTG
EoMumix3	GCCTCYATTTCGTCGAATCCS
JIG-337	CTGGCAGGAAAATGCAACCT
JIG-338	GCAATTGCGCAAGCTTAATG
JSR01dsR	GGGTTCGAAATCGATCGGGATA
JIG-368	CGGAGTCATTCTTGCTCTTCT
JGp3dsL	ACCCGACCGGATCGTATCGG
JIG-369	AGGACGAGACCTTAGTCCTTAT

Table S3: Summary of EM-seq libraries

source tissue	library name	SRA run accession	150nt read pairs	mCHH of lambda spike-in control
mdr1 mutant 15-DAP endosperm	m1-EN	SRR18289836	47014301	0.21%
mdr1 mutant 15-DAP endosperm	m2-EN	SRR18289835	45701409	0.19%
mdr1 mutant 15-DAP endosperm	m4-EN	SRR18289834	44436792	0.25%
wild-type 15-DAP endosperm	w1-EN	SRR18289859	45875343	0.20%
wild-type 15-DAP endosperm	w2-EN	SRR18289858	51596713	0.19%
wild-type 15-DAP endosperm	w3-EN	SRR18289847	47357875	0.22%
mdr1 mutant 15-DAP embryo	m1-EM	SRR18289830	51981508	0.19%
mdr1 mutant 15-DAP embryo	m2-EM	SRR18289857	46147134	0.36%
mdr1 mutant 15-DAP embryo	m4-EM	SRR18289856	65684146	0.15%
wild-type 15-DAP embryo	w1-EM	SRR18289833	39632962	0.18%
wild-type 15-DAP embryo	w2-EM	SRR18289832	41814310	0.18%
wild-type 15-DAP embryo	w3-EM	SRR18289831	49590739	0.16%
mdr1 mutant mature endosperm	mdr1-endo-10_EM-seq_rep1	SRR15725961	36817426	0.28%
mdr1 mutant mature endosperm	mdr1-endo-35_EM-seq_rep2	SRR15725960	26612972	0.05%
mdr1 mutant mature endosperm	mdr1-endo-36_EM-seq_rep3	SRR15725949	28459057	0.04%
wild-type mature endosperm	wt-endo-21_EM-seq_rep1	SRR15725938	35145084	0.33%
wild-type mature endosperm	wt-endo-28_EM-seq_rep2	SRR15725937	30343910	0.14%
wild-type mature endosperm	wt-endo-44_EM-seq_rep3	SRR15725936	34218216	0.03%
mdr1 mutant premeiotic tassel	mdr1_J500m_EM-seq_rep1.2	SRR15725935	40010980	0.20%
mdr1 mutant premeiotic tassel	mdr1_J500m_EM-seq_rep2	SRR15725934	18296892	0.09%
mdr1 mutant premeiotic tassel	mdr1_J500m_EM-seq_rep3	SRR15725933	24344086	0.30%
wild-type premeiotic tassel	wt_J500s_EM-seq_rep1	SRR15725932	21951025	0.17%
wild-type premeiotic tassel	wt_J500s_EM-seq_rep2	SRR15725959	20640290	0.23%
wild-type premeiotic tassel	wt_J500s_EM-seq_rep3	SRR15725958	28257660	0.37%

Table S4: Summary of small RNA-seq libraries

source tissue	library name	SRA run accession	mapped 20-25nt reads
mdr1 mutant 15-DAP endosperm	m1-EN-sRNA	SRR18289849	19019110
mdr1 mutant 15-DAP endosperm	m2-EN-sRNA	SRR18289848	23095644
mdr1 mutant 15-DAP endosperm	m4-EN-sRNA	SRR18289846	15272395
wild-type 15-DAP endosperm	w1-EN-sRNA	SRR18289852	10781412
wild-type 15-DAP endosperm	w2-EN-sRNA	SRR18289851	12580298
wild-type 15-DAP endosperm	w3-EN-sRNA	SRR18289850	7650061
mdr1 mutant 15-DAP embryo	m1-EM-sRNA	SRR18289842	20728999
mdr1 mutant 15-DAP embryo	m2-EM-sRNA	SRR18289841	6980356
mdr1 mutant 15-DAP embryo	m4-EM-sRNA	SRR18289840	16381870
wild-type 15-DAP embryo	w1-EM-sRNA	SRR18289845	12255179
wild-type 15-DAP embryo	w2-EM-sRNA	SRR18289844	24472485
wild-type 15-DAP embryo	w3-EM-sRNA	SRR18289843	21938914
mdr1 mutant premeiotic tassel	mdr1_J500m_small_RNA-seq_rep1	SRR15725951	16802824
mdr1 mutant premeiotic tassel	mdr1_J500m_small_RNA-seq_rep2	SRR15725950	20520237
mdr1 mutant premeiotic tassel	mdr1_J500m_small_RNA-seq_rep3	SRR15725948	11922885
wild-type premeiotic tassel	wt_J500s_small_RNA-seq_rep1	SRR15725947	11143536
wild-type premeiotic tassel	wt_J500s_small_RNA-seq_rep2	SRR15725946	24948764
wild-type premeiotic tassel	wt_J500s_small_RNA-seq_rep3	SRR15725945	18451175
