# Supplementary material for: The maize gene maternal derepression of r1 encodes a DNA glycosylase that demethylates DNA and reduces siRNA expression in the endosperm
Source: Plant Cell. 2022 Jul 1;34(10):3685–701. doi: 10.1093/plcell/koac199 (PMC9516051; doi:10.1093/plcell/koac199)
Supplement: koac199_Supplementary_Data [file tpc21.00848.PeerReviewReport.pdf › koac199_Supplementary_Data/tpc21.00848.PeerReviewReport.pdf]

# The maize gene *maternal derepression of r1* encodes a DNA glycosylase that demethylates DNA and reduces siRNA expression in the endosperm

Jonathan I. Gent, Kaitlin M. Higgins, Kyle W. Swentowsky, Fang-Fang Fu, Yibing Zeng, Dong won Kim, R. Kelly Dawe, Nathan M. Springer, and Sarah N. Anderson

*Corresponding author: Jonathan I. Gent, gent@uga.edu*

**Review timeline:**

| | | |
|---|---|---|
| **TPC2021-RA-00848** | Submission received: | Sept. 30, 2021 |
| | 1st Decision: | Nov. 11, 2021 *decline with external review* |
| **TPC2021-RA-00848R1** | 1st Revision received: | Mar. 29, 2022 |
| | 2nd Decision: | May 5, 2022 *accept with minor revision* |
| **TPC2021-RA-00848R2** | 2nd Revision received: | May 24, 2022 |
| | 3rd Decision: | June 7, 2022 *acceptance pending, sent to science editor* |
| | Final acceptance: | June 27, 2022 |

**REPORT:** (The report shows the major requests for revision and author responses. Minor comments for revision and miscellaneous correspondence are not included. The original format may not be reflected in this compilation, but the reviewer comments and author responses are not edited, except to correct minor typographical or spelling errors that could be a source of ambiguity.)

**TPC2021-RA-00848 1st Editorial decision – *decline with external review*** **Nov. 11, 2021**

Thank you for choosing to send your manuscript to The Plant Cell. Your submission has been evaluated by members of the editorial board as well as expert reviewers in your field, and we regret to inform you that we are not able to recommend publication of this manuscript. We have not made this decision lightly. We have had input from multiple scientists and have solicited post-review comments as well. Our present policy is to offer streamlined decisions and to not advise on the direction of the work by requesting extensive modifications or substantial additional experiments.

While two of the reviewers are broadly in support of publication, each of them had significant issues that include both problems with the methodology and a requirement for new data. A third reviewer found this work of issue primarily to the maize community, as many of the observations made here have been made before in other systems. Many of the methodological questions can be addressed. For instance, a better description of the phenotypic effects of the double mutants on the gametophyte, more statistical analysis of the data, a more careful description of the genetic experiments and a description of changes in methylation at the *r1* locus. However, there are comments that require additional data. For instance, it is unclear why endosperm DMRs were compared with RdDM targets in the tassel rather than directly testing for enrichment of RdDM sites among mdr1 DMRs. The depth in the coverage of DNA methylation is inadequate; given that this is an analysis of the effects of the mutant on methylation, proper sequencing depth is essential. Finally, any evidence of subfunctionalization of the homeologs would be of interest and distinct from what has been seen in Arabidopsis. Collectively, the reviewers agreed that these data are potentially important to the extent that the study provides us with novel insights that arise from novel effects of these mutants on a genome rich in transposable elements, but that this would require additional evidence.

Therefore, we cannot recommend resubmission. Nevertheless, we do hope that the comments below are helpful to you in preparing a revision for another journal.

[Decision altered following successful appeal]

-------------------------------------------------------------------------- Reviewer comments:

[Reviewer comments shown below along with author responses]

Reviewer comments and **author responses**:

Reviewer #1:

The manuscript by Gent et al., "The maize gene *maternal depression of r1* (*mdr1*) encodes a DNA glycosylase with maternal and paternal fertility functions", describes the cloning of the maize gene responsible for the activation of the maternal *R1* gene, causing the red kernel phenotype. The analysis of *mdr1* endosperm methylome gains insights into the function of MDR1 DNA glycosylase.

The Maize maternal *R* is uniformly active and yields solidly pigmented endosperm. When maternal *R* is absent, non-uniform and weak expression of paternal R causes a mottling pattern. Kermicle subsequently reported that mottling can occur in the presence of maternal R in a mutant he called *mdr* (for *maternal derepression of R*). In this study, the authors used a bulk segregant sequencing approach to map the location of *mdr1* to the long arm of chr4 and identified the causal mutation in a DNA glycosylase gene homologous to the Arabidopsis DNA glycosylase gene (DME).

In Arabidopsis, maternal inheritance of a mutant *dme* allele causes seed failure whereas seeds inheriting a paternal *dme* allele are normal and viable. Also, in certain ecotypes, *dme* paternal sperm are less efficient that wild-type sperm during reproduction. The rice OsROS1a (or DNG702) was shown to be the functional counterpart of DME in rice, demethylating the vegetative (VC) genome and presumably the central cell (CC) genome, due to the extensive overlap of DMRs in VC and in endosperm. These results suggest that Arabidopsis and rice gamete companion cell (VC and CC) demethylation is an evolutionarily conserved phenomenon in dicot and monocot. Although the biological function the companion cell demethylation remains to be fully elucidated, reinforcing gamete DNA methylation via mobile siRNAs and de novo methylation appears to be one common function between rice and Arabidopsis.

One reason why *mdr1* escaped genetic identification might be due to redundancy with the *dng102* gene. Genetic results infer that double mutant *mdr1* and *dng102* causes gametophyte failure in both male and female gametophytes. However, *mdr1/mdr1* homozygous seeds are viable which permitted the authors to interrogate the endosperm methylome.

Although much was learned from the study of DME and ROS1 in Arabidopsis and in rice, respectively, TEs and repeats make up a much smaller portions of the Arabidopsis or rice genomes compared with the maize. Consequently, many new features and functions of the DNA demethylation system can be learned by studying maize genes that we might not have otherwise been revealed by studying Arabidopsis or rice. Although I find the study interesting and could be significant, the data and analysis presented are often descriptive, preliminary, and should be further refined. This is particularly true in the genetic and phenotypic study of the mutant phenotypes.

**Response: First, we thank the reviewer for a thoughtful and thorough review, which led to many improvements in the revised manuscript. In regard to genetic and phenotypic study, we have made several improvements and additions in the revision. We have demonstrated that a second *mdr1* mutant allele, EMS4-06835d, is also unable to transmit maternally or paternally unless a functional Dng102 allele is present. We also now demonstrate that double mutant *mdr1 dng102* megagametophytes are fertilization-competent but die early in development, while the defect in double mutant microgametophytes makes them fertilization-incompetent. We include more phenotypic descriptions now in the main Figures 1 and 2 of the paper, along with data on expression of *mdr1*, *dng102*, and two other related maize dng genes across tissues; data on their maternal/paternal expression bias in endosperm; and data on their relationships to other DNG proteins in sorghum, rice, and Arabidopsis (including *DEMETER*).**

Some of the methylome analysis can also be further extended to improve the characterization of molecular phenotypes caused by *mdr1*.

**Response: In the revised manuscript, we have refined and extended the data and analysis using a new set of methylomes from 15-DAP endosperm and paired embryos, both wild-type and *mdr1* mutant. In fact, we have added 170 billion nt of EM-seq read coverage to the new analysis (see Supplemental Table S3). The new data allowed us to identify nearly twice as many endosperm DMRs. Analysis of these DMRs confirmed all our previous conclusions,**

**but with even more robust support, and allowed us to identify a Helitron family that is enriched for DMRs. We have also prepared and analyzed small RNA data from the same samples, which demonstrates that normal MDR1 function inhibits rather than promotes siRNA accumulation.**

Specific comments for the authors to address are listed below.

Line 9, "Demethylation of transposons in the central cell" is a more appropriate statement.

**Response: It is not clear whether initial demethylation in central cell or the subsequent demethylated state in endosperm is what "can activate expression of nearby genes and cause imprinted gene expression in endosperm". Thus we think it better to not invoke central cells in this sentence.**

Lines 57-60, this was first reported in Hsieh et al., 2009

**Response: We have added Hsieh et al. 2009 to the references here.**

Line 65, Hsieh et al., 2011 should be included here.

**Response: We have added Hsieh et al. 2011 to the references here.**

Line 69, this statement is not correct. Hsieh et al 2009 showed endosperm CHG and CHH are higher than arial tissues.

**Response: We should have clarified that non-CG methylation is reduced relative to embryo, not arial tissues. However, as this point probably distracts from the main point that Polycomb Repressive Complex2 (PRC2) promotes non-CG methylation in endosperm, we have removed that part of the sentence. We have also added the Hsieh et al. 2009 references here.**

Figure 1B:

i. The G3987A mutation is different from C to T nucleotide change in the main text (Line 135).

**Response: We have clarified in the main text that the C to T change is relative to the genome reference sequence, resulting in a G to A change in mRNA.**

ii. The dng102-Q235 whereas *mdr1s* were not labeled with amino acid # change. It would be better to be consistent throughout the manuscript.

**Response: We are hesitant to introduce more nomenclature. We could name the Kermicle allele in a way that indicates it is a string of amino acid changes followed by a premature stop codon, but instead we just exactly describe the allele in the main text, and clarify that unless indicated otherwise, all experiments are done using the Kermicle allele. We had to name the *dng102* allele something since it has never been referenced before, which is why we call it dng102-Q235.**

The *R1* locus methylation profiles in the assayed tissues should be shown as this is the de facto MDR1 target gene.

**Response: We had not included *r1* methylation originally because we only included the ten assembled chromosomes in our analysis, and *r1* is on a scaffold because of its difficult-to-assemble multicopy status. We have now included r1 methylation in Figure 3A (CG methylation only) and Supplemental Figure S3 (all methylation contexts). While the analysis is limited because of multicopy sequence and N gaps near and in the gene, there is clearly differential methylation in the promoter and 5' UTR of r1 in the *mdr1* mutant.**

Figure S1 is confusing, is the color designation switched?

**Response: Yes, this was a mistake. It is now corrected.**

Lines 127-128, "The long arm of chromosome 4, however, was nearly identical to W22 (Supplemental Figure S2)." This was not obvious on Fig S2, probably because the length of chromosome was not indicated? Fig S2 also says "These regions do not include the *mdr1* region on the top of 4L. This is all very confusing.

**Response: Our apologies for a confusing figure. We have merged Figure S1 and S2 into a new Figure S1, added a schematic illustration to it, labeled and color coded better, and clarified both in the main text and figure legend how to interpret it.**

Line 134, the EMS allele is in B73 right? Please indicate in the text.

> **Response: Correct, the EMS allele is in B73, and we have now included that in the results section too rather than just in the methods section.**

Lines 139-143, kernel images of cross result should be shown.

> **Response: We have added images of kernels to Figure 2.**

Lines 148-150, please show expression in a sup figure.

> **Response: We now show expression across tissues in Figure 1D and show maternal expression ratios in endosperm in Figure 1E. Surprisingly, *mdr1* is biallelically expressed, though with a maternal preference, while *dng102* is biallelically expressed but with a paternal preference.**

Lines 151-153, the Kapazoglou et al tree did not include all maize paralogs. It would be nice to generate a new phylogenic tree for this purpose.

> **Response: We have generated a tree for maize DNGs, as well as sorghum, rice, and Arabidopsis. See Figure 1B. This confirms the close relationship of MDR1 and DNG102, and identifies two other distinct DNGs in maize, all of which have clear homologs in sorghum.**

Lines 157-158, add mutation info (e.g., C703T, Q###>*).

> **Response: We have added the Q235 detail into the sentence.**

Lines 159-161, the phenotype picture and genetic data should be shown.

> **Response: The new Figure 2 includes phenotypes and genetics.**

The Ds-GFP experiment, selection of Ds-GFO alleles tdsgR128F11 and tdsgR153F01 were not adequately explained (i.e., how are they linked to each gene) in the Method section, main text, or in the legend of Fig S3.

> **Response: Our apologies for poorly explained Ds-GFP experiments and results. We have added a schematic of the genetics in Figure 2A, and modified the Results and Discussion sections of thr main text and the figure legend to clarify these points.**

On page 8 and in Fig S3, what has been presented is more of a qualitative description, not a quantitative presentation of actual results. The authors should present results from each direction of the cross, phenotype and genotype results, and perform proper statistical analysis. Some of this part of the results should be shown in the main figure as this is one important conclusion of the study.

> **Response: The entire Figure 2 is now dedicated to the Ds-GFP experiments and analysis. We also include a more thorough explanation in the main text of the paper, including raw numbers of fluorescent and non-fluorescent kernels. We include P-values for the new defective kernel frequencies, which are significantly different depending on the direction of the cross (P-value < .00001)**

Line 169-170, I have trouble reaching the same conclusion as the authors based on this alone, without seeing the genetic results mentioned in line 160, since single *dng102* mutant allele alone does not cause an obvious phenotype (line 159) and *mdr1/mdr1* kernels are viable.

> **Response: The schematics in Figure 2 now explain the logic behind the conclusions on the block to mutant transmission, the paternal pre-pollination defect, and the maternal post-pollination defect.**

If this result is substantiated, I wonder if they authors can analyze the pollen pool to gain at least some physiological insight into the phenotype of *mdr1/dng102* pollen (germination defect or abnormal pollen grains, etc).

> **Response: We certainly would like to expand our study to include developmental/morphological characterization of mutant pollen. Advice from maize pollen experts indicates these experiments in maize are not trivial to do properly. We are currently trying to obtain funding for a salary for someone to do these experiments.**

Similarly, on the female side, crossing the GFP-linked, double heterozygous female with wildtype male pollen resulted in high frequency of empty kernel positions, and the genetic data (ratio of empty positions) should be

presented along with statistical analysis. If possible, it is desirable to assess whether the defect is manifested in the gametophyte or at post fertilization.

> **Response: The new data in Figure 2D and E showing and quantifying early defective kernel phenotypes demonstrates that the maternal defect manifests at least partially post-fertilization.**

EM-seq info on Table S2, please provide conversion rate information.

> **Response: We have added mCHH values from the unmethylated lambda DNA spike-in for every sample as an additional column in the new supplemental Table S3. These values indicate all samples had conversion rates of greater than 99.99%. This is further supported by the genome-wide low levels of mCHH, as indicated by methylation in DMR control regions in Figure 4A-C and supplemental Figure S7 and the near-zero mCHH in gene transcription start sites in supplemental Figure S3A.**

In Arabidopsis, *dme* endosperm (counterintuitively) causes a reduced in non-CG methylation compared with wild-type endosperm. Since *mdr1* endosperm methylome was the first monocot demethylase mutant endosperm methylome, it would be highly informative to show gene and TE whole genome metaplots among different assessed tissues, and if necessary, separate CG and CHG DMRs to see if assessing CG DMR alone influence any conclusion obtained from CG/CHG combined DMRs.

> **Response: We looked into the possibility of the *mdr1* mutant having an opposite or negligeable on mCHG as mCG before deciding to require increase in both as a defining feature of DMRs. The general trend for mCHG was to increase I the mutant, though not as consistently as mCG. At a genome-wide level this is supported by the methylation in DMR control regions shown in Figure 4A-C and supplemental Figure S7. These control regions are primarily made up of TEs. We also included a meta-gene methylation plot in supplemental Figure S3A, as requested.**

Lines 210-212. "Indeed, 78.5% of the DMRs from the comparison with *mdr1* were within a kilobase of a DMR identified from the comparison with tassel or leaf." Please present direct overlap information and the rationale why the authors used 1kb proximity, and what this means biologically. I would expect a high level of complete overlap rather than vicinity. I could understand if it is due to sequencing depth differences, but it is not possible to compare the same 200bp windows across all comparisons and a 1kb is a safe distance to capture related DMRs. I am asking the authors to explain why they use 1kb vicinity as criteria. I believe the authors want to say that leaf and tassel can be good proxies for *mdr1* endosperm. So, in theory, DMRs between wt endo vs mdr1, wt endo vs leaf, wt endo vs tassel, the 3 DMR sets should be highly overlapping. Since their DMRs were defined in 200-bp windows, and due to low coverage, they cannot easily merge 200-bp into larger DMRs. So, they used 1kb as "acceptable gaps" to say >78% DMRs are indeed close to each other. I think they need to describe this at greater details and more convincingly.

> **Response: The summary the reviewer provides here is quite good, and we would borrow it for the main text, except it is no longer necessary. Instead of focusing our methylation and siRNA analysis on WT endosperm vs. WT tassel DMRs as a proxy, we now focus directly on WT endosperm vs. mdr1 endosperm DMRs.**

Fig 2B, it would be informative to give N= for each annotated feature and # of DMR overlap with them.

> **Response: Indicating the number of annotated features would be informative if sufficient detail were also added to contextualize those numbers, which is difficult to do. For example, although knob180 is a 180 bp tandem repeat, it makes up ~50 Mb of the genome, most of which is not included in the assembly. Of the knob180 that is included in the assembly, most lacks sufficient polymorphism to map EM-seq reads too. However, we have added a Supplemental Table S2 listing the exact number of DMRs and control regions that overlap with each annotated feature.**

Fig 2C, please explain how enrichment # (y-axis) is calculated.

> **Response: Enrichment is the proportion of DMRs that overlap each element by at least 50% of their length (100 bp) divided by the proportion of control regions that do. We have put this in the new figure legend.**

Lines 246-247, "In contrast, the whole set of gene ends were about 50% less likely to overlap DMRs than the rest of the genome was." Where is the data?

**Response: This data is now in Figure 3C.**

Fig 4 and the siRNA section on page 13, the endosperm and embryo siRNA data were generated from B73 whereas the DMR of this study were from W22. The authors should show B37 siRNA data can represent siRNA population in W22. This is needed for an important conclusion of this study that DMRs are not enriched for siRNA.

**Response: This was a misunderstanding. All small RNA and EM-seq data we analyzed were from W22. On that note, the new small RNA data generated for the revision is from the same exact tissue sources as the EM-seq data rather than from the prior Fu et al (2018 publication), so this is clearer now.**

Also, there is a small enrichment of 24-nt siRNAs in endosperm and a larger enrichment in embryo compared with eligible regions (Fig 4 D) in the euchromatin DMRs. It is possible the embryo siRNAs are influenced by hypomethylation in endosperm.

**Response: We have now directly tested the impact of demethylation by MDR1 on siRNAs. MDR1 clearly inhibits accumulation of 24-nt siRNAs in endosperm, and it may have a slight inhibitory effect in embryo as well (Figure 4G-I and supplemental Figure S8). The enrichment of 24-nt in DMRs relative to eligible regions (control regions) is likely a consequence of DMRs having some overlap with regions undergoing RdDM, as RdDM is also enriched near genes in endosperm and plant tissues.**

Fig 5, please give the gene numbers for each class of genes in each comparison as well as # of MEGs and PEGs.

**Response: We have added the numbers of genes to Figure 5.**

Fig 5D, needs a color scale and the color choice is not easily visible and not visually intuitive, perhaps the authors can try drawing line plot with fractional mCG value across or a delta line plot between tassels and endosperm?

**Response: We have removed this plot entirely. The new Figure 3A with methylation of *r1* makes it somewhat redundant. We have not changed the color choice, however, as the CGmapTools software does not have options for colors.**

Line 347, Schoft et al 2011 PNAS should be included here.

**Response: we have added Schoft et al. 2011 to the references here.**

Line 357, Hsieh et al., 2011 PNAS should be included here.

**Response: We have added Hsieh et al. 2011 to the references here. On that note, it would be helpful if the reviewer would explain all cases where specific additional references are requested. This is a rich field with multiple possible papers to cite, and we have mainly limited citations to one or several of the most informative ones or ones with particular historical value. It would have been helpful to know something of the reviewer's insights into the importance of the specific citations requested.**

Page 17, top section, perhaps the authors should discuss the observation that 24-nt siRNA are enriched in developing embryo loci corresponding to endosperm DMRs (Fig 4D, are these loci overlap with RdDM loci?)

**Response: We have added the following sentence to the discussion to address this: "Related to this, the preferential demethylation near genes in endosperm suggests the possibility of interaction with RdDM, which is strongly enriched near genes (Gent et al. 2014; Fu et al. 2018). However, the low mCHH and siRNA abundance in demethylated regions in endosperm suggest otherwise."**

<u>Reviewer #2:</u>

The authors of this manuscript identified a mutation in the DEMETER-LIKE glycosylase Mdr1 to be responsible for the mottling phenotype in maize kernels in the presence of a maternal R allele. The authors identified Mdr1 and its close homolog Dng102 as the two major glycosylases expressed in the endosperm. Using segregation analysis of linked Ds-GFP markers, the authors show that one functional allele of either Mdr1 or Dng102 is required for proper male and female fertility and/or development of seeds. The authors further show that Mdr1 is responsible for hypomethylation in CG and CHG contexts in euchromatin regions that are likely not targeted by RdDM. The authors claim that demethylation of the genome does not lead to increased formation of siRNAs, supporting recent work

showing that DNA demethylation is unlikely to enforce silencing in the embryo (DOI: 10.1186/s13059-021-02358-3). Last, the authors found that DMRs in endosperm are particularly enriched at imprinted genes, in particular at the 5' end of MEGs. However, since most expressed genes overlapping DMRs in the endosperm are not imprinted, the authors inferred that Mdr1 may not have a major effect on gene imprinting.
While the identification of the causal mutation of the mdr1 mutant is interesting, the manuscript does not really provide major new insights into the functional role of DNA glycosylases in the endosperm.

**Response: First, we thank the reviewer for the helpful and insightful comments, which led to an improved revised manuscript. Regarding the concern about lack of major new insights, we would like to point out that prior expectations for endosperm DNA glycosylases based on published work in both Arabidopsis and rice is that we would find MDR1 primarily demethylates short transposons near genes and the edges of long transposons deeper in heterochromatin. In addition, one would expect that MDR1 activity in or near genes would be a strong indicator of imprinted gene expression. We have found both expectations to be wrong. Admittedly this does not reveal what the functional role of MDR1 actually is, but correcting misconceptions is often a necessary step toward further advances. That said, in the revised manuscript, we have clearly demonstrated something new regarding function of MDR1 in that it inhibits rather than promotes siRNAs in endosperm (Figure 4G-I and Supplemental Figure S8. Related to that, and in contrast to hypotheses based on work in Arabidopsis and rice, it also does not lead to increased methylation in embryo (Figure 4D-F and Supplemental Figure S7).**

A major shortcoming of the manuscript is that the developmental consequences of loss of Mdr1 and Dng102 for male and female fertility were not investigated. Based on failed transmission of both mutant alleles the authors show that both genes are essential for male and female fertility; however, a characterization of the developmental defects in mdr1 dng102 mutants would be required to understand the cause for the transmission defect.

**Response: While carrying out detailed developmental phenotyping of pollen and endosperm is beyond the scope of the current project, we did show that the male fertility defect manifests prior to fertilization (Figure 2B,C). Now in the revised manuscript, we have also shown that the female fertility defect manifest post-fertilization as small defective kernels (Figure 2D,E)**

In addition, the poor coverage of the DNA methylation data strongly limits the conclusions.

**Response: In the revised manuscript, we have extended the data and analysis using a new set of methylomes from 15-DAP endosperm and paired embryos, both wild-type and *mdr1* mutant. In fact, we have added 170 billion nt of EM-seq read coverage to the new analysis (see Supplemental Table S3). The new data allowed us to identify nearly twice as many endosperm DMRs. Analysis of these DMRs confirmed all our previous conclusions, but with even more robust support, and allowed us to identify a Helitron family that is enriched for DMRs.**

Furthermore, inferring a direct effect of MDR1 activity on genomic imprinting and gene expression requires to be validated by transcriptome data of mdr1 mutant endosperm.

**Response: We would like to argue that the statistically robust correlation between known imprinted genes and TEs with the mdr1 DMRs we have identified is sufficiently strong evidence that demethylation in endosperm can facilitate imprinted maternal gene expression (Figure 5). This argument is further supported by the fact that DNA glycosylases in Arabidopis and rice are already well established to function similarly.**

Last, the manuscript is very difficult to follow because of the numerous comparisons of different types of DMRs, which are largely not required and rather distract from the main point of the manuscript, the functional role of Mdr1.

**Response: We have reduced the number of comparisons of different types of DMRs to just two in the main figures: WT endosperm vs. WT embryo DMRs, and WT endosperm vs. mdr1 mutant endosperm DMRs. To further avoid distraction, we reduce this to just WT endosperm vs. mdr1 mutant endosperm DMRs for all detailed methylation and siRNA expression analyses. We include additional comparisons with premeiotic tassel and mature endosperm in supplemental materials.**

Other comments:

The mapping strategy and corresponding crosses should be visualized in a Supplementary figure.

**Response: we have added a schematic depiction of mapping strategy and crosses as supplemental Figure S1A.**

L 148 ff: The expression data should be shown.

**Response: We now show expression across tissues in Figure 1D and show maternal expression ratios in endosperm in Figure 1E. Surprisingly, mdr1 is biallelically expressed, though with a maternal preference, while dng102 is biallelically expressed with a paternal preference.**

A Suppl. figure showing conservation of DME, Mdr1 and Dng102 would be helpful.

**Response: We have added a protein tree as well as percent identities as Figure 1B. This confirms the close relationship of MDR1 and DNG102, and identifies two other distinct DNGs in maize, both of which have clear homologs in sorghum.**

Based on the description, the treatment of the replicates to identify DMRs is unclear. The authors state that the replicates were merged, but then refer to "pair of merged CGmaps". It should be clarified what exactly has been done. Also the conversion rate should be indicated as supplementary information.

**Response: We have now clarified in the methods section that replicates were combined prior to calling DMRs (to maximize sequence coverage for DMR calling). Regarding conversion rates, we have added CHH methylation values from the unmethylated lambda DNA spike-in for every sample as an additional column in the new supplemental Table S3. These values indicate all samples had conversion rates of greater than 99.99%. This is further supported by the genome-wide low levels of mCHH, as indicated by methylation in DMR control regions in Figure 4A-C and supplemental Figure S7 and the near-zero mCHH in gene transcription start sites in supplemental Figure S3A.**

Screenshots of the identified DMRs should be provided to estimate the quality of the data.

**Response: We have included *r1* as an example differentially methylated region in *mdr1* mutant endosperm. See Figure 3A (CG methylation only) and supplemental Figure S3 (all methylation contexts). This is technically not in our list of DMRs because it is on a genomic scaffold and we only used the ten chromosomes for DMR analysis, but this locus is particularly relevant because it explains the mdr1 pigmentation phenotype.**

Figure 2 and corresponding paragraph. The data are presented without statistical treatment, limiting the value of the conclusions. Furthermore, the paragraph is very difficult to follow, it would be important to clearly highlight which conclusions can be made based on the analyses. It is unclear why RdDM regions are included in the analysis, if this is relevant, it should be clearly phrased.

**Response: We have completely redone this figure (now Figure 3) and rewritten the paragraph to clarify how we draw our conclusions. We made use of entirely new methylomes, from 15-DAP endosperm and 15-DAP embryo, both WT and mdr1 mutant. These new data are higher coverage and identify about twice as many DMRs as previous endosperm DMRs. They reproduce and confirm the results from mature WT and mdr1 endosperm and WT tassel and WT leaf used in the original manuscript. Due to the large numbers of control regions and DMRs overlapping each class of genetic element, all enrichments/depletions shown in Figure 3B are highly significant (P-values < .0001 for differences between controls and DMRs for all categories of repetitive elements in the figure). However, we have also carried out an analysis of specific TE families including some with as few as three copies, and for these we present FDR stats (supplemental Figure S5C).**

Figure 2A. The overlap of identified DMRs should be graphically visualized.

**Response: We have removed Figure 2A. It was a useful figure before to justify using WT endosperm vs. WT tassels DMRs for detailed small RNA and methylation analysis, but now we use WT endosperm vs. WT mdr1 endosperm.**

Figure 2B: The authors used tassel CHHm data to identify RdDM regions, but the comparison is then made with endosperm regions targeted by Mdr1. Therefore, identifying RdDM-targeted regions in the endosperm would be more relevant.

**Response: We now use mCHH data from 15-DAP endosperm to identify RdDM regions, which we now call mCHH regions. We only show these regions in Figure 4I and Supplemental Figure 8 in relation to small RNAs, as including RdDM regions with the DMR enrichment plots was distracting from the main point.**

Figure 3 requires improvement. It is unclear what is the difference between developing endosperm and endosperm. It would be advisable to only include one developmental stage that corresponds to the mdr1 sample.

**Response: We have completely redone Figure 3 (now Figure 4) using the one developmental stage: 15-DAP endosperm and embryo. We have moved mature endosperm and premeiotic tassel to Supplemental Figure S7.**

Also the figure caption is unclear, what is a typical methylation profile?

**Response: We have removed this from the new legend.**

Figure 4; if the authors plan to identify demethylation-dependent sRNAs, it is unclear why the authors did not focus on MDR1-dependent DMRs but instead investigated endosperm-tassel DMRs.

**Response: We now focus on MDR1-dependent DMRs (WT endosperm vs. mdr1 endosperm DMRs) instead of endosperm-tassel DMRs.**

Figure 5B, C: Are differences statistically significant?

**Response: We have tested for significant differences between the imprinted and non-imprinted categories in Figure 5B. The 9-fold increased frequency of MEGs over non-imprinted genes overlapping with DMRs is significant for both sets of DMRs. We indicated this in the new figure. None of the endosperm gene expression categories in Figure 5C are significantly different from each other, meaning endosperm expression in general is not correlated with DMRs.**

Figure 5D: A screenshot of *mdr1* endosperm should be included.

**Response: We have removed Figure 5D because methylation of r1 is more relevant than the myb155 gene. See Figure 3A for *mdr1* mutant and WT (CG methylation only) and Supplemental Figure S3 (all methylation contexts).**

Line 308ff: The indicated overlap refers to which DMRs?

**Response: We have clarified in the text that the overlap enrichment is true for both sets of DMRs.**

Since the manuscript started with the regulation of *R1* by Mdr1, DNA methylation data of r1 in mdr1 endosperm should be provided.

**Response: We did not include *r1* methylation originally because we only included the ten assembled chromosomes in the analysis, and *r1* is on a scaffold because of its difficult-to-assemble multicopy status. We have now included *r1* methylation in Figure 3A (CG methylation only) and Supplemental Figure S3 (all methylation contexts). While the analysis is limited because of multicopy sequence and N gaps near and in the gene, there is clearly differential methylation in the promoter and 5' UTR of *r1*.**

Minor comments:

Line 33: Since DME is also expressed and functions in pollen, as stated by the authors, the statement that it is primarily active in the female gametophyte should be revised. The paper that first demonstrated a function of DME in pollen should be included (doi: 10.1073/pnas.1105117108)

**Response: We have edited the sentence to clarify that DME is primarily active in central cell relative to endosperm specifically. We have also added the Schoft et al. 2011 citation to the next sentence, where we introduce the pollen vegetative cell.**

Line 64: Global reduction of CG methylation in the central cell was not confirmed by Park et al., 2016 (doi: 10.1073/pnas.1619047114).

**Response: Our intent was to point out that met1 is downregulated in central cell, not that mCG is reduced in central cell. Since met1 is replication-coupled, its downregulation in central cell would not be expected to significantly effect mCG in the central cell. Instead, it could set the stage for mCG reduction during early cells divisions of the endosperm. Since this would be wordy to explain and what we had written was misinterpreted, we just removed the reference to central cell altogether from this section.**

Line 87: The model that demethylation in the central cell/endosperm promotes methylation in the embryo was recently questioned (doi: 10.1186/s13059-021-02358-3), which should be acknowledged.

**Response: Chakraborty et al. 2021 is an excellent paper, which we already had cited in the discussion section. It does not directly test the model for glycosylase driven siRNA expression, however, so we are keeping this citation in the discussion section rather than further complicating the introduction section.**

Reviewer #3:

This manuscript describes the cloning and characterization of mdr1, a DEMETER homolog in maize. The key conclusions are that 1) MDR1 is one of two DNA glycosylases demethylating endosperm and that these demethylases are also required in both female and male gametophytes, 2) the demethylated regions are not necessarily enriched for transposable elements, and 3) many, but not all, regions demethylated in endosperm are associated with imprinted gene expression. The analysis is done to a high standard, however there is little novelty in the conclusions, most of which have been previously demonstrated in Arabidopsis and/or rice.

> **Response: First, we are grateful for the reviewer's comments on specific points in the manuscript, which helped us to improve it. In regard to overall significance of our conclusions, we would like to ask for a revaluation based on the responses we provide below.**

As described in the introduction, DEMETER-type glycosylases are known to be essential in reproductive development in Arabidopsis and rice. Similarly, Gehring et al 2009 Science have already demonstrated that most demethylated regions in the endosperm are not associated with imprinting (and most imprinting regions are associated with demethylation). Recapitulating these findings in maize is important, but unlikely to be of broad interest.

> **Response: There appears to have been a misunderstanding here. We agree that it is well established that most endosperm DMRs are not near imprinted genes. The novelty we are describing is that even most genes that overlap endosperm DMRs are not imprinted. This is an important point because it suggests an unidentified role in endosperm demethylation that is unrelated to distinguishing maternal and paternal alleles.**

There are also several prior papers that include a thorough assessment of DNA methylation its link to imprinting in maize.

> **Response: Prior maize imprinting and methylation papers did not analyze DNA glycosylase mutants and were consequently limited to analysis of loci which could be distinguished by maternal and paternal SNPs in hybrid endosperm. They also did not examine siRNAs in endosperm.**

In Lines 88-96, the authors lay out the differences between maize and rice or Arabidopsis, including lack of CMT2, a high number of transposons, and different endosperm development. While these differences support studying whether loss of DEMETER activity causes the same phenotype in maize as observed in rice and Arabidopsis, they also support the conclusion that this research will be of limited interest outside of the maize community.

> **Response: As an alternative perspective, consider that maize being different from two model plants with smaller genomes does not make it an outlier. In fact, in many respects, maize has a more representative genome and endosperm structure, especially compared to other cereals of agricultural importance (e.g., wheat, barley, sorghum, rye, etc). In addition, even if that were not the case, maize would be worth understanding solely because of its own agricultural importance. We are not dismissing the importance of Arabidopsis or rice as models: in fact it is because of the foundation of work in Arabidopsis that we were able to immediately interpret the significance of the mdr1 candidate gene and know to examine methylation in endosperm as first priority. If the reviewer would like further convincing of the significance of this work, we have added in the revision an experiment that has never been done in Arabidopsis or rice: a direct test the effect of a DNA glycosylase mutant on siRNA expression and on methylation in paired mutant and wild-type embryos and endosperm. The result is clear: DNA glycosylase activity inhibits rather than promotes siRNA expression.**

Detailed comments:

The genetics to demonstrate failure of the *mdr1 dng102* double mutant gametophytes is very nice. Kudos!

> **Response: That is good to hear, because we thought it was a clever use of the Ds-GFP collection. Based on comments from other reviewers, we have further improved and extended the description of the mdr1 dng102 double mutant analyses in the revised manuscript in Figure 2.**

It is essential that conversion rates for the EM-seq are included to demonstrate that the libraries are of sufficient quality for analysis. Conversion should be >99%.

**Response: We have added CHH methylation values from the unmethylated lambda DNA spike-in for every sample as an additional column in the new supplemental Table S3. These values indicate all samples had conversion rates of greater than 99.99%. This is further supported by the genome-wide low levels of mCHH, as indicated by methylation in DMR control regions in Figure 4A-C and supplemental Figure 76 and the near-zero mCHH in gene transcription start sites in supplemental Figure S3A.**

I was glad to see careful comparison against regions with enough sequence depth for analysis ("eligible regions"), rather than against the genome as a whole. However, given that a DMR must have a 20% reduction in CG and CHG methylation, then regions that don't have this level of methylation in WT endosperm cannot become DMRs in mdr1 endosperm. (Similarly, regions without that level of methylation in tassel or leaf cannot become DMRs in WT endosperm.) Since nearly all of the analysis focuses on hypoDMRs, it would be helpful to use the methylated subset (as in line 242-244) when assessing enrichment or depletion of genomic features.

**Response: We have done exactly this, now more extensively than in the original submission. See supplemental Figure S6 which shows enrichment for DMRs relative to the set of control regions with at least 20% mCG and mCHG in at least one of the two samples in each comparison (methylated control regions). This reveals that DMRs are enriched relative to the set of methylated control regions inside genes, even in transcription start sites. Interpreting this result is complicated though because regions that are methylated in both CG and CHG contexts in maize gene exons are usually annotation errors. We are currently carrying out a detailed study of gene body methylation in maize, which will have a lot more on this topic. In regard to depletion of DMRs from most repetitive elements, comparing to methylated control regions produced nearly identical results, which is not surprising since most control regions in repetitive elements are methylated.**

One idea that I found somewhat novel was the conclusion that demethylation of repetitive DNA (ie, transposons) is not the "major function" of MDR1 and its paralog. This derives from the observation that transposons are not enriched among DMRs (whether defined by comparing tissues or comparing genotypes). This conclusion is at odds with observations from other systems and needs further investigation. The authors here use a strict definition for DMRs. Would the same conclusion hold with more relaxed criteria? Or if endosperm was assayed at a different developmental stage? What might feasibly explain the difference between this observation and the many published reports that repetitive elements are the main target of endosperm demethylation?

**Response: We have not done a reanalysis of methylation data from endosperm of other plants using carefully defined control regions to measure enrichment. This is something that would be worth doing. We can say confidently, however, that any differences with conclusions from the prior literature are not due to endosperm staging though, because our new analysis of 15-DAP endosperm gives nearly identical results as our original analyses of mature endosperm in terms of depletion of DMRs from repetitive elements.**

I'm a little unclear on why the manuscript repeatedly describes MDR1 demethylation as "distinct from RdDM" (eg, lines 215, 273-274). The manuscript does not test whether RdDM is required for MDR1 activity, and does not even directly test for enrichment of RdDM sites among mdr1 DMRs. Rather, enrichment of features at sites of RdDM are compared with enrichment of features at DMRs - a somewhat indirect comparison.

**Response: We had put a lot of emphasis on comparing RdDM regions and endosperm DMRs because of our prior misconception that endosperm DMRs would correspond to RdDM in other tissues. This was a miner point relative to the main conclusions of the paper, and the RdDM regions were distracting to people, so we mainly removed them from the analyses. The exception is in Figure 4I and Supplemental Figure 8, where it is still worthwhile to demonstrate that mCHH is a good indicator of siRNAs, even in endosperm, and that the DMRs are only about a quarter as likely as mCHH regions to have siRNAs in endosperm. Note that we now call them mCHH regions rather than RdDM regions.**

Also, why are RdDM sites identified in tassel? Why not use the metric of >20% CHH in WT endosperm, where the authors have more data?

**Response: We now use mCHH in WT endosperm instead of WT tassel.**

The section starting in line 277 tests the idea that demethylation of the endosperm might trigger siRNA production. While interesting, by assessing DMRs between the tassel and WT endosperm, this section moves away from the role of MDR1.

**Response: We now use the DMRs between WT endosperm and mdr1 endosperm, which indeed connects more directly to the role of mdr1. See Figure 4G-I and Supplemental Figure 8.**

Line 61, 373: *Brassica rapa*, not *napa*

**Response: We have corrected this.**

**TPC2021-RA-00848R1 2nd Editorial decision – *accept with minor revision* May 5, 2022**

We have received reviews of your manuscript entitled "The maize gene *maternal derepression of r1* (*mdr1*) encodes a DNA glycosylase that demethylates DNA and reduces siRNA expression in endosperm." On the basis of the advice received, the board of reviewing editors would like to accept your manuscript for publication in The Plant Cell. This acceptance is contingent on revision based on the comments of our reviewers. In particular, please consider the following:

Although both of the reviewers were satisfied with the revisions, each of them had some minor concerns prior to publication

**TPC2021-RA-00848R2 2nd Revision received May 24, 2022**

Reviewer comments and **author responses**:

Reviewer #2:

In this revised version of the manuscript the authors added additional data to (i) substantiate their previous findings and to (ii) generate new insights into the function of MDR1. This version is a clear improvement compared to the previous one and the findings worth to be reported after addressing some additional comments. My comments mainly aim to avoid misconceptions (comment 1), to link this manuscript with previously published data (comment 2) and to further improve readability (comment 3).

Specific comments:

1. The authors argue that because the majority of genes that overlap demethylated regions are not imprinted, genomic imprinting is not the main function of MDR1 in the endosperm. However, the authors find that maternally-expressed imprinted genes preferentially overlap with MDR1 demethylated regions, which suggests a clear requirement of MDR1 in the regulation of imprinted genes, very similar to the function of DNA glycosylases in Arabidopsis and rice. I therefore consider it well possible that main function of MDR1 is to regulate imprinted genes; just because not all genes flanking demethylated regions are imprinted, does not refute this hypothesis. Demethylation is likely a prerequisite for imprinting; it is necessary, but not sufficient. I would strongly suggest to tone down the claim that genomic imprinting is not the main function of MDR1 in the endosperm and to consider alternative scenarios.

**Response: It is not only the fact that most genes that overlap with DMRs are not imprinted that lead us to propose a main function for demethylation other than imprinting, it is also the fact that over half the DMRs are not even near euchromatin. However, we agree with the Reviewer's statement, "Demethylation is likely a prerequisite for imprinting; it is necessary, but not sufficient". In addition, while imprinting may not be the main function, it may be the most important function because of the effect on a few key endosperm genes. We have added a couple sentences to the last paragraph of the discussion to clarify this:**

**"This suggests that demethylation is important for imprinting but not sufficient. It also suggests that although genomic imprinting of specific genes may be essential for endosperm development, genomic imprinting is not the main effect of DNA demethylation in endosperm."**

2. Previous work of the authors (doi: 10.1371/journal.pgen.1009491) identified the DHH00002 helitron family to have many maternally expressed members and to be enriched for PHE1 binding sites. It would be relevant to link these findings with the data shown in this manuscript. Here, the authors find that Helitrons from the DHH00002 family overlap DMRs more frequently than expected; I therefore suggest testing whether maternally expressed DHH00002 members are demethylated and whether the expression status correlates with the presence of PHE1 binding sites.

**Response: The previous paper already demonstrated that imprinting status of DHH00002 elements was not correlated with presence of PHE1 motifs. However, we have now added an analysis to the revised manuscript showing that DHH00002 elements with PHE1 motifs are more likely to overlap DMRs than those without (Supplemental Figure 5C). We have also added an analysis showing that about a third of the imprinted MEG type TEs are members of the DHH00002 family (Supplemental Figure 5D). Thus PHE1 motifs appear to be connected to DNA demethylation, and DHH000002 elements frequently have imprinted expression, but the connection between PHE1 motifs, DHH00002 elements, and imprinting is complicated.**

3. The definition and use of hypoDMRs in relation to *mdr1* is highly confusing. The *mdr1* mutation causes hypermethylation in comparison to wt, it would therefore be more logic to define DMRs between mutant and wt as hyperDMRs. This confusion is also reflected in the text, line 305 "The *mdr1* mutant reduces both methylation..."; in fact the *mdr1* mutation increases rather than reduces methylation.

**Response: We have corrected the mistake from "reduces" to "increases". Thanks for catching that. The reason we use the term endosperm "hypoDMRs" rather than "hyperDMRs" is that it allows for a clearer comparison between WT vs. mdr1 endosperm DMRs and WT endosperm vs. WT embryo DMRs. In both cases, WT endosperm is what is hypomethylated.**

Reviewer #4:

The authors have addressed the issues and questions I have raised. The revised manuscript, along with the new analysis, makes the paper much improved and easier to comprehend. The main conclusions of the paper are clearly supported by the data. Since the majority of the maize genome consists of TEs and repeats, this study complements what we have learned from Arabidopsis and rice and provides new insights into this still enigmatic epigenetic process essential for angiosperm reproduction. The redundancy with DNG102, the expression post-fertilization in embryo and endosperm, the enrichment of Helitrons in DMRs, the high methylation level in the DMRs across development, and higher DMR associated siRNA accumulation in *mdr1* are all fascinating features of MDR1 that demand attention and further investigation. The redundancy between MDR1 and DNG102 also allows a direct assessment of how the loss of MDR1 activity affects embryo methylation and siRNA accumulation which was not yet possible to study in rice or Arabidopsis. Overall, the study provides compelling evidence that MDR1 and DME likely employ distinct molecular mechanisms that reflect their respective genome composition.

I am satisfied with the current manuscript, except for this very minor issue below that I believe was not correct:

Line 208, "...., it was significantly lower in maternal mutant crosses compared..." It refers to the frequency of dek, shouldn't it be higher when the mutant is the maternal parent?

**Response: Thanks for catching that mistake. We corrected it from "lower" to "higher".**

**TPC2021-RA-00848R2 3rd Editorial decision – *acceptance pending*** **June 7, 2022**

We are pleased to inform you that your paper entitled "The maize gene maternal derepression of r1 (mdr1) encodes a DNA glycosylase that demethylates DNA and reduces siRNA expression in endosperm" has been accepted for publication in The Plant Cell, pending a final minor editorial review by journal staff.

**Final acceptance from Science Editor** **June 27, 2022**
